# Maple samara flight is robust to morphological perturbation and united by a classic drag model

**DOI:** 10.1038/s42003-024-05913-3

**Published:** 2024-03-01

**Authors:** Breanna M. Schaeffer, Spencer S. Truman, Tadd T. Truscott, Andrew K. Dickerson

**Affiliations:** 1 Mechanical, Aerospace, and Biomedical Engineering, University of Tennessee, Knoxville, TN USA; 2 Department of Mechanical Engineering, Physical Science and Engineering Division, King Abdullah University of Science and Technology, Thuwal, 23955 Kingdom of Saudi Arabia

**Keywords:** Biophysics, Plant physiology

## Abstract

Winged, autorotating seeds from the genus *Acer*, have been the subject of study for botanists and aerodynamicists for decades. Despite this attention and the relative simplicity of these winged seeds, there are still considerable gaps in our understanding of how samara dynamics are informed by morphological features. Additionally, questions remain regarding the robustness of their dynamics to morphological alterations such as mass change by moisture or area change by damage. We here challenge the conventional approach of using wing-loading correlations and instead demonstrate the superiority of a classical aerodynamic model. Using allometry, we determine why some species deviate from interspecific aerodynamic behavior. We alter samara mass and wing area and measure corresponding changes to descent velocity, rotation rate, and coning angle, thereby demonstrating their remarkable ability to autorotate despite significant morphological alteration. Samaras endure mass changes greater than 100% while maintaining descent velocity changes of less than 15%, and are thus robust to changes in mass by moisture or damage. Additionally, samaras withstand up to a 40% reduction in wing area before losing their ability to autorotate, with the largest wings more robust to ablation. Thus, samaras are also robust to wing damage in their environment, a fact children joyfully exploit.

## Abstract (web summary)

Winged, autorotating seeds from the genus Acer can autorotate despite significant morphological alteration, enduring mass changes >% while maintaining descent velocity changes of <%. A classical aerodynamic model well predicts descent velocity.

## Introduction

**Fig. 1:**
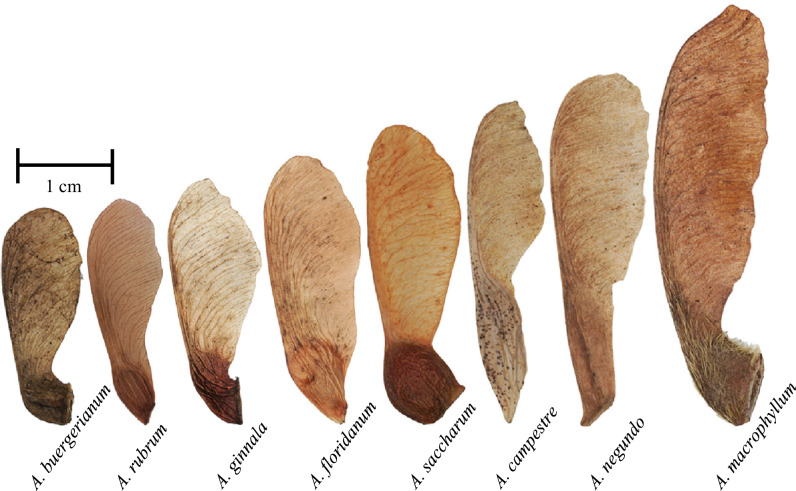
Samara species tested in this study. The average span of the species increases from left to right.

The mesmerizing aerial performance of the samara seeds, known as autorotation, has long captured the curiosity of both nature enthusiasts and scientists alike. Beyond their visual appeal, the autorotating flight of maple seeds, also called samaras, serves an ecological purpose. Samara-bearing trees have developed the ability to produce winged seeds as a means to enhance proliferation. The samara wings enable the seeds to leave the parent trees, extending their reproductive reach and enhancing the chances of successful establishment by increasing the time from tree to ground^1–5^. The subjects of this study, fruits of the genus *Acer* (*A*.) seeds vary in size, mass, and shape (Fig. 1), yet maintain a tight band of descent velocities across species.

**Fig. 2:**
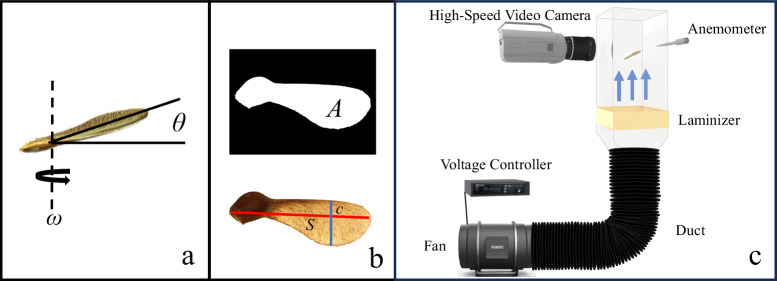
Measured parameters and experimental setup. **a** Schematic of samara in flight with coning angle *θ* and angular velocity *ω* labeled. **b** Visual representation of area *A*, span *S*, and chord *c* taken from image analysis. **c** Experimental setup.

To increase the time from tree to ground, samaras slow their descent by autorotation that generates lift and exposes more of the samara area to oncoming air, increasing drag in descent. Samara seeds have developed a sophisticated interplay between morphological factors to facilitate autorotation. The winged structure of the seed increases the surface area exposed to the air, resulting in an aerodynamic drag force preventing the seed from falling rapidly^6^, an ability that remains intact even in the presence of a strong crosswind^5^. The majority of samara mass is concentrated towards the heavier nutlet^7,8^, such that on one side of the center-of-mass, the samara is denser and rounded. On the other side of the center-of-mass is the high-aspect-ratio, and higher drag wing. The result is that samaras autorate with the nutlet down to create a coning angle *θ*, shown in Fig. 2a. Aerodynamic and centrifugal forces of the spinning samara achieve equilibrium, imparting stability to both the angle of attack and coning angle during descent^4,9,10^.

**Fig. 3:**
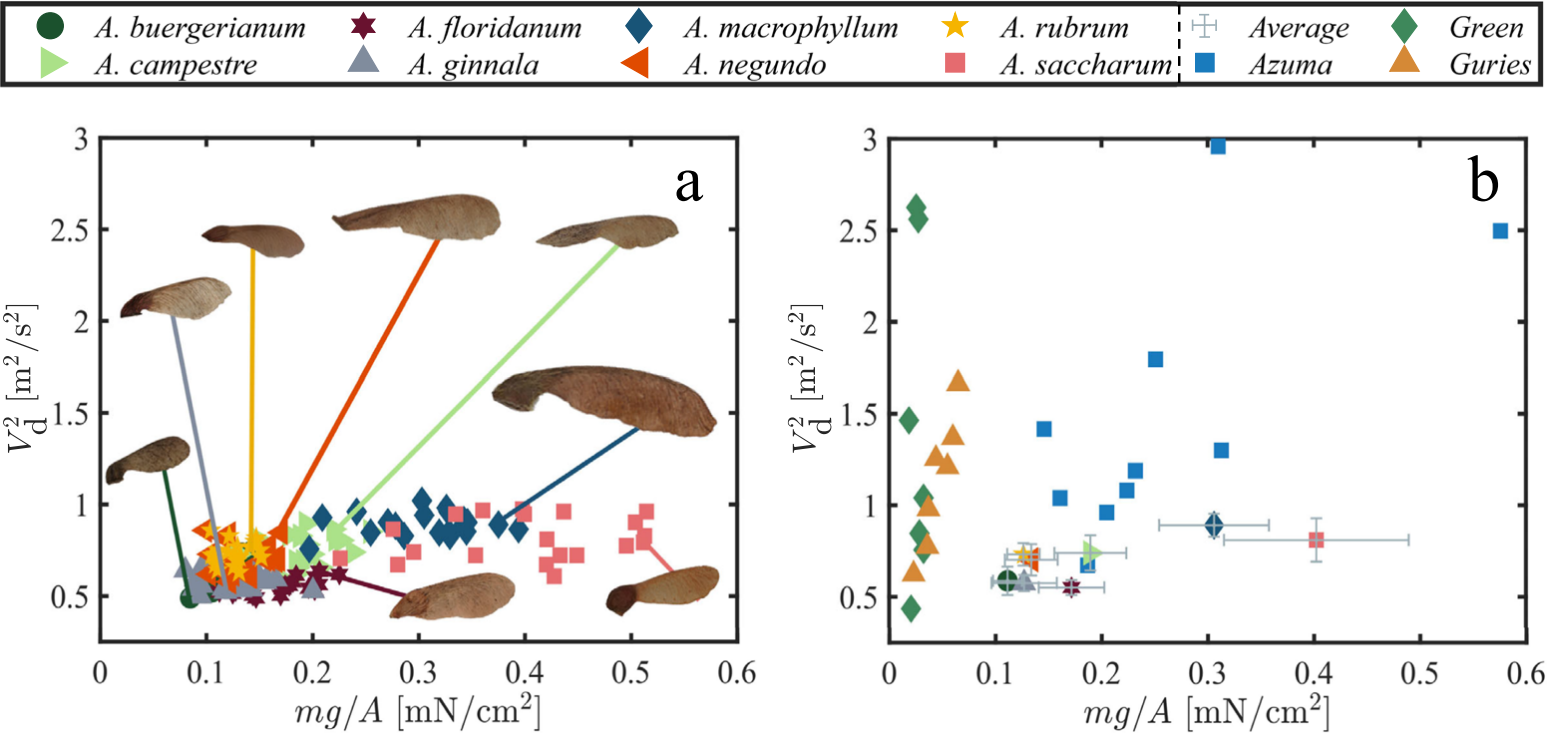
Relation between descent velocity and wing loading. Relation between descent velocity *V*_d_ and wing loading *mg*/*A* from (**a**) this study, and (**b**) previous studies [Azuma and Yasuda^15^, Green^11^, Guries et al.^16^] with matched axis scales for comparison. Points in (**b**) with error bars are averages of species taken in this study. Error bars represent one standard deviation.

The relation between the dynamics of autorotation and the morphological features of samaras is complex, has been the focus of multiple previous studies^4,8,11–14^, and is not yet fully characterized. Efforts to improve modeling accuracy by increasing complexity have not provided tools by which multiple samara species can be compared. Among the various aerodynamic variables measured, descent velocity *V*_d_ stands out as the most investigated quantity. The standard approach to link *V*_d_ to morphological characteristics, is what has been termed wing loading^11^,

1 $${V}_{{{{{{{{\rm{d}}}}}}}}}^{2}\, \sim \,mg/A,$$

where *m* is samara mass, *A* is plan-view area, and *g* is the acceleration due to gravity. We present the relation between ${V}_{{{{{{{{\rm{d}}}}}}}}}^{2}$ and *mg*/*A* for the samaras of this study shown in Fig. 3a. Wing loading suitably assesses the loading profile of an individual wing across diverse speeds but proves less effective as a metric for comparing distinct wing shapes between *Acer* species–a distinction we explore in greater detail.

Furthermore, as we compile and analyze samara studies conducted by various researchers^11,15,16^, a striking lack of agreement between terminal velocity and wing loading becomes apparent, as depicted in Fig. 3b. This recurring pattern strongly suggests that additional factors should be considered when forming a relationship that relates samara size to its rate of descent.

In this study, we conduct measurements of the morphological and dynamic characteristics of eight distinct *Acer* species. A photograph of samaras lying flat on a tabletop is taken for image processing in MATLAB to measure geometric dimensions. Samara area *A* is that of the nutlet and wing as seen in Fig. 2b. The span *S* is defined as the longest dimension of the entire samara and chord *c* is the maximum samara dimension perpendicular to the span line. We explore samara dynamics with high-speed videography and apply a classical aerodynamic drag model to determine how samaras of different species can be scaled as a unified group. Samaras are released into the wind tunnel, as schematized in Fig. 2c, and wind speed is adjusted to facilitate stable hovering. From video analysis, we measure angular velocity *ω* and *θ* as illustrated in Fig. 2a. We film three replicates of each individual samara.

## Results and discussion

### Aerodynamic drag unifies samara species

**Fig. 4:**
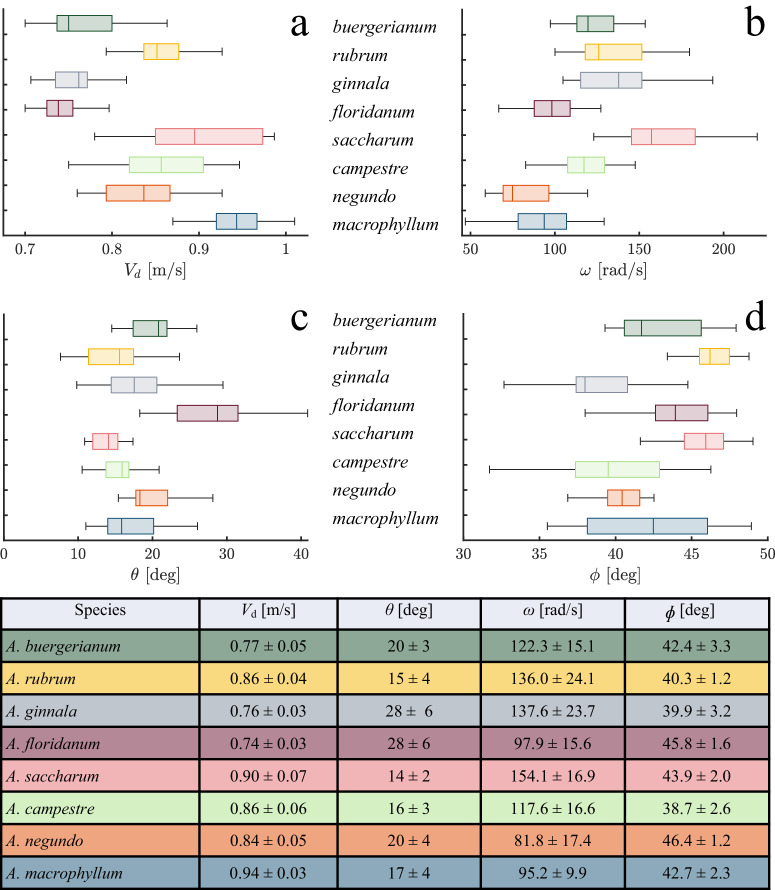
Dynamic measurements of test species. **a** Descent velocity *V*_*d*_, **b** angular velocity *ω*, **c** coning angle *θ*, and **d** pitch angle *ϕ*. The table contains dynamic measurement averages and standard deviations. *n* = 20 for each species.

We begin exploring the slow descent of samaras by filming hovering, stably autorotating samples in a wind tunnel, and measuring the dynamic quantities recorded in Fig. 4. We find our samaras have an average terminal velocity *V*_d_ = 0.83 ± 0.08 m/s (*n* = 160 biologically independent samples), values which appear to have no apparent correlation with coning angle *θ* = 117.8 ± 29. 1° (*n* = 160), or angular velocity *ω* = 18.8 ± 4.6 [rad/s] (*n* = 160, see Fig. S2). The angle of attack varies by wing location and thus is tabulated at 0.75*S* from the nutlet tip such that $\phi =\arctan ({V}_{d}/0.75S\omega )=42.5\pm 3.{2}^{\circ}$ (*n* = 160), and is nicely schematized by Lentink et al.^17^. Included as supplementary plots in Figs. S2–S4, our exploration of isolated dynamic variable relationships did not reveal any notable correlations. Instead, considering dynamic variables in functional groups is more fruitful.

**Fig. 5:**
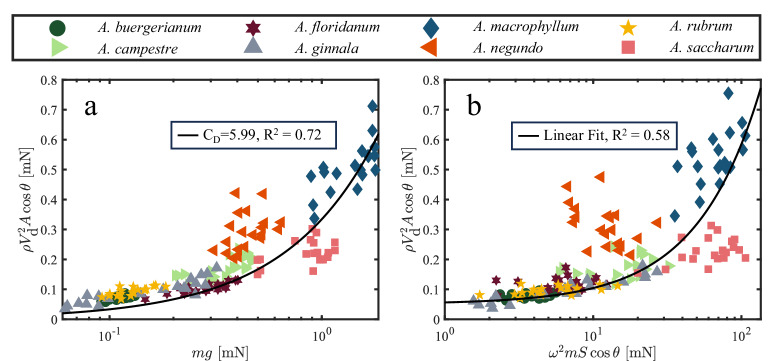
Salient forces acting on centroid during descent. **a** Drag force to samara weight *mg* and (**b**) drag force to centrifugal force.

At its core, the idea of ‘wing loading’ as plotted in Fig. 3 is an expression of aerodynamic drag by considering the samara a free-falling blunt body. In fact, the form drag and lift generated by an autorotating samara act against the direction of motion. We, therefore, lump the two effects into a simple force ${F}_{D}=\rho {V}_{{{{{{{{\rm{d}}}}}}}}}^{2}A\cos \theta$. From a vertical force balance, one would expect

2 $$mg \sim \frac{1}{2}{C}_{{{{{{{{\rm{D}}}}}}}}}\rho {V}_{{{{{{{{\rm{d}}}}}}}}}^{2}A\cos \theta ,$$

where *g* = 9.81 m/s^2^ is the acceleration due to gravity, *C*_D_ is a drag coefficient, *ρ* = 1.23 g/cm^3^ is the air density, and $A\cos \theta$ is the projected area of the seed in the direction of vertical motion (Fig. 2c). This approach is similar to the descent factor proposed by Lentink et al.^17^. We plot $\rho {V}_{{{{{{{{\rm{d}}}}}}}}}^{2}A\cos \theta$ versus seed weight *mg* for all tested samaras in Fig. 5a. Each data point in Fig. 5 is comprised of three replicates. The slope of the line of best fit in Fig. 5a, with a correlation coefficient *R*^2^ = 0.72, indicates *C*_D_ = 5.99 across all test species. A unifying drag coefficient allows for the comparison of individual seed performance to the bulk, and lumping dynamic variables as done in Fig. 5a supplies a superior correlation between descent and weight than the wing loading of Fig. 3 (*R*^2^ = 0.35). Lentink et al.^17^ also combined the drag and lift forces in the vertical direction through a descent factor. Here, we add the coning angle *θ* which incorporates the effective lift force in the vertical direction. This important distinction will be shown in “Samara morphology perturbation” to scale all samaras better as it connects the lift force to *ω*, *V*_d_, and *mg*.

Emerging from the drag plot of Fig. 5a are two species that notably depart from the trend line. *A. Negundo* samaras have greater drag than might be expected for their weight, a possible consequence of mass distribution across a long, flat body (Fig. 1). We deem *A. Negundo* samaras to be ‘underweighted’ in this context. *A. Saccharum* samaras fall more quickly than expected for their weight, or are ‘overweighted’, a likely consequence of their bulbous seed mass (Fig. 1). The concepts of over- and under-weighted do not materialize from tabulated dynamic measurements (Fig. 4), but do from simple morphological measurements presented in “Samara morphology distinguishes species”.

The consideration of samaras as falling blunt bodies fails to capture the rotational dynamics occurring in the plane normal to descent. The autorotation of samaras is a direct result of the air momentum passing over the wings^4^, to produce a centrifugal force about the samara centroid. Such rotation increases the aforementioned *C*_D_, thus slowing the samara *V*_d_. Therefore, the expected relation between descent and rotation is not abundantly clear^18^, and samara rotation is underexplored in literature. Previous studies^4,15^ analyzed rotational velocity within species, but lacked correlations across different species. We explore descent and rotation by plotting their respective associated forces in Fig. 5b. It should be noted that we do not expect the relation between drag force and centrifugal force to be linear, but the slope of the line of best fit, 5.3 × 10^−3^ (*R*^2^ = 0.58), in Fig. 5b provides a metric to compare the magnitude of the forces acting on the samara centroid. Centrifugal forces dominate those acting at the centroid. Underweighted seeds convert relatively less momentum from the passing air to angular momentum than overweighted seeds.

In the following sections of this paper, we will explore through morphological measurements, deviations from the Fig. 5 trend lines, and determine through morphological perturbation, if a simple drag model continues to describe seeds that are burdened with additional mass or reduced mass by damage.

### Samara morphology distinguishes species

In this section, we explore the drag and centrifugal force trends described in “Aerodynamic drag unifies samara species” by considering simple morphological measurements and their respective relationships.

#### Samara morphology is diverse

**Fig. 6:**
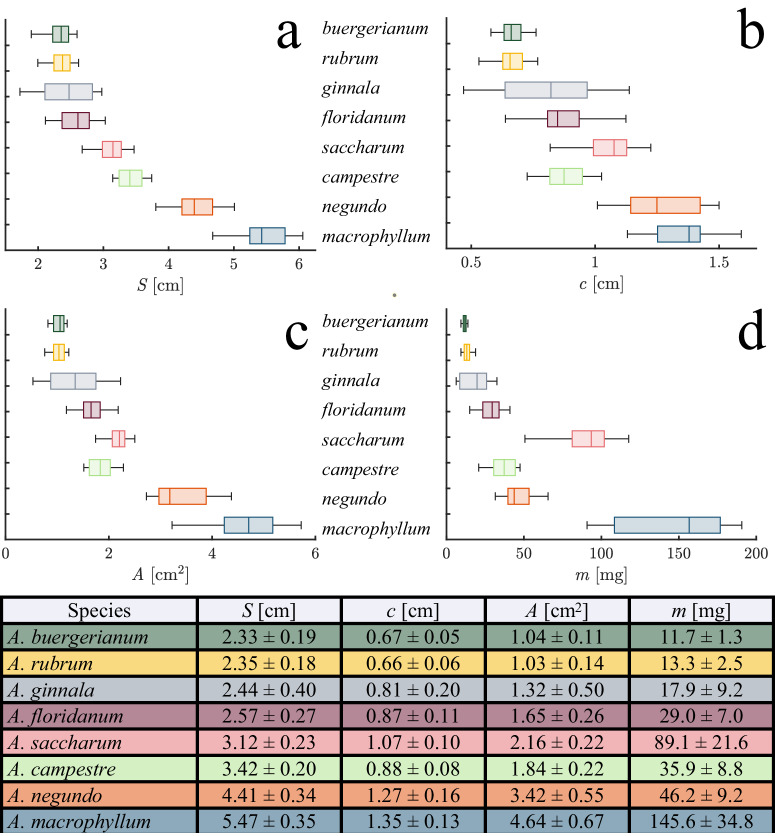
Morphological measurements for tests species. **a** Span *S*, **b** chord *c*, **c** area *A*, and **d** mass *m*. The table values are averages and standard deviations. *n* = 20 for each species.

It is a wonder that samaras within and across species descend with such similar speeds despite their large variation in shape, scale, and proportion (Fig. 1). Such a variation likely contributes to researchers’ struggle to succinctly characterize samara mechanics. Our specimens range in mass *m* = 6.3 − 19.1 mg, span *S* = 17.2 − 60.6 mm, and area *A* = 0.53 − 5.72 cm^2^, as recorded in Fig. 6. *A. saccharum* and *A. buergerianum* possesses sizeable, quasi-spherical nutlets, whereas *A. negundo* and *A. campestre* are equipped with elongated, flat nutlets. *A. macrophyllum* showcases trichomes adorning the nutlet area, while *A. ginnala* nutlets culminate in a distinct, sharp, flat edge. Samara wings likewise have broad variations in both aspect ratios, curvature, and texture. We measure the chord-span ratios of the triangular wings of *A. ginnala* and the rectangular wings of *A. floridanum* to be 0.33 and 0.34 respectively. By comparison, the slender wings of *A. campestre* have a chord-span ratio of 0.25. Wing proportions govern lift production and the mass moment of inertia, and will have an influence on agility^18–20^.

We arrange the order of species in Fig. 6 according to Fig. 1, that of increasing span. The intraspecific variability rampant in *Acer* samaras can be attributed to genetic diversity, environmental influences, selective pressures acting on populations, or the position of the seed within the tree canopy^21–24^. Notable intraspecific variation is observed in the chord and span of *A. Ginnala* with the smallest measured specimen achieving 41% the chord and 58% the span of the largest *A. Ginnala* specimen, as shown in Fig. 6a, b, respectively. *A. Macrophyllum* has the largest intraspecific variation in area and mass, with the smallest specimen weighing just 48% that of the largest, as shown in Fig. 6c, d. While tabulated values of morphological measurements are useful for assessing variation, the relation between physical dimensions is difficult to ascertain.

#### Samaras are two-dimensionally allometric

Within and across allometric species *A* ~ *L*^2^ and *m* ~ *L*^3^, where *L* is some characteristic length. Here we take the characteristic length of a samara to be its span *S* and likewise assume the other dominant length *c*. In this scenario, the chord is an alternative characteristic length scale. To satisfy allometry, it thus follows that

3 $$A \sim {m}^{2/3} \sim {S}^{2} \sim {c}^{2} \sim cS.$$

**Fig. 7:**
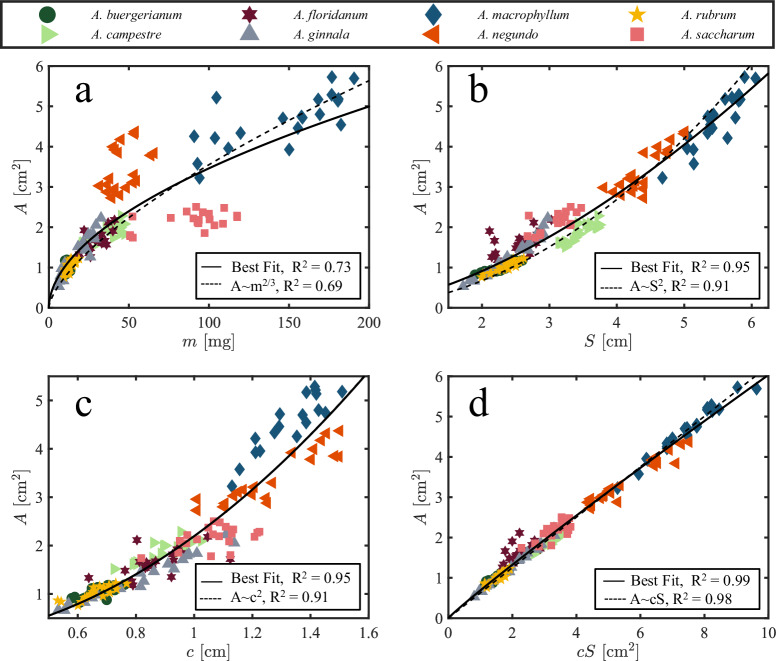
Allometric relations. **a** area *A* vs mass *m*, **b** *A* vs the square of span *S*^2^, **c** *A* vs the square of chord *c*^2^, and **d** *A* vs *cS*.

We explore the allometric predictions in Eq. (3) in Fig. 7, and report our results in turn. We plot *A* versus *m* with a best-fit power law in Fig. 7a. The allometric prediction yields a correlation coefficient *R*^2^ = 0.69. A best fit scaling for all our specimens, *A* ~ *m*^0.52^, *R*^2^ = 0.73, a notable departure from Eq. (3). However, if we had not included *A. Negundo* and *A. Saccharum* in our study, our best fit would yield *A* ~ *m*^0.57^. The origins of the underweighted (*A. Negundo*) and overweighted (*A. Saccharum*) species become apparent from Fig. 7a, a classification that is difficult to ascertain from traditional treatment by wing loading of samaras (Fig. 3). The mass-to-area ratios of Fig. 7a suggest that *A. Saccharum* samaras are naturally closer to the limit of mass they can support than are *A. Negundo* samaras, a hypothesis we test in “Samara morphology perturbation”.

Deviation from interspecific allometric predictions by *A. Negundo* and *A. Saccharum* appears to be caused only by nutlet mass. Otherwise, all our tested species are wonderfully allometric. We plot *A* versus *S* in Fig. 7b, yielding a best-fit scaling of *A* ~ *S*^1.63^, accompanied by a correlation coefficient of *R*^2^ = 0.95 and a modest improvement over the predicted scaling correlation coefficient, *R*^2^ = 0.91. The plot of *A* versus *S* supports span as an appropriate characteristic length scale. However, the best-fit power law of *A* ~ *c*^1.99^, *R*^2^ = 0.95 is remarkably close to Eq. (3), and is plotted in Fig. 7c. The allometric prediction yields a correlation coefficient of *R*^2^ = 0.91. Chord is thus the single best length to characterize samara size. Lastly, the plot of *A* against *cS* exhibits a best-fit scaling of *A* ~ *cS*^0.94^, yielding an exceptional correlation coefficient of *R*^2^ = 0.99 (Fig. 7d). While the *A* versus *m* plots distinctly differentiate between overweighted and underweighted seeds, the other allometric plots fail to showcase these distinctions explicitly. Within these plots, both *Acer Saccharum* and *Acer Negundo* demonstrate geometric correlations that align with allometric predictions.

While samaras do exhibit allometric relationships, deviations and interspecific variations in these relationships highlight the robustness, or perhaps lack of fine-tuning in their adaptations. Deviations from allometric predictions, particularly stemming from the mass-to-area relationship as illustrated in Fig. 7a, underscore the uniqueness of each species’ evolutionary trajectory. Key questions arise: How do external alterations in mass and area provided by environmental challenges influence individual seeds and their aerodynamic behavior? Does a simple drag model capture the behavior of modified samaras?

### Samara morphology perturbation

#### Samaras are robust to mass addition

Suspended samaras are susceptible to environmental moisture such as rainfall and dewfall, a fate shared with foliage^25^ and resting insects^26,27^. As samaras turn from green to brown, or in cycles of drought, one would expect their moisture content, and thus mass, to fluctuate. Some nutlets contain a seed while others are empty^28^. It follows, therefore, that samara flight is robust to some range of mass alteration. Our chosen species for mass augmentation are the over- and underweighted species identified in “Aerodynamic drag unifies samara species”, and two additional species for comparison, the largest test species, *A. macrophyllum* and the smallest, *A. buergerianum*. We augment the mass of three specimens per species by dunking the nutlet in melted wax, where the number of dunks controls the amount of mass addition. The addition of wax contributes a <5% increase in *A* (Fig. S5). Mass is added in subsequent flight tests until the samaras cannot levitate in our tunnel. We also subtract mass from one specimen per species by shaving mass from the nutlet in a manner similar to that done by Varshney et al.^7^. Mass is reduced in subsequent flight tests until the samaras cannot levitate in our tunnel. We provide movies of weighted and unweighted samaras alongside their unaltered states hovering in our tunnel in Movies S1 and S2, respectively.

**Fig. 8:**
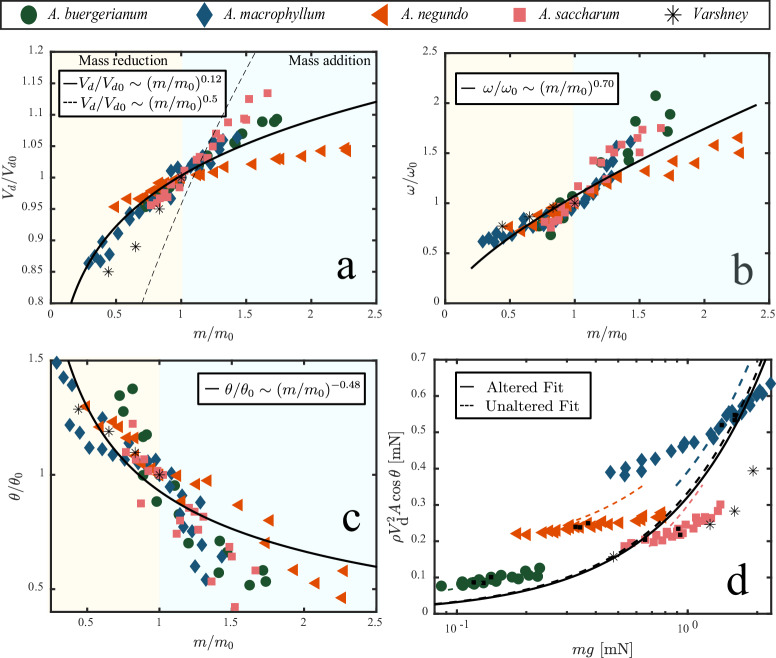
Samaras are robust to mass addition. **a** Reduced velocity *V*_*d*_/*V*_d0_ vs reduced mass *m*/*m*_0_, **b** reduced angular velocity *ω*/*ω*_0_ vs reduced mass *m*/*m*_0_, **c** reduced cone angle *θ*/*θ*_0_ vs reduced mass ratio *m*/*m*_0_ vs, and **d** drag force to samara weight *mg*.

We plot reduced velocity *V*_d_/*V*_0_ vs reduced mass *m*/*m*_0_ in Fig. 8a up to the point where modified samaras no longer auto-rotate, where *V*_d0_ and *m*_0_ are the unmodified descent velocity and mass for each samara on test, respectively. Our largest samara, *A. macrophyllum*, tolerates the least amount of relative mass addition before failure at *m*/*m*_0_ ≈ 1.3 but performs best in relative mass reduction down to *m*/*m*_0_ ≈ 0.3. The overweighted samaras of *A. saccharum* (largest mass to area ratio Fig. 7a) are the most sensitive to mass addition, $V_{{{\mbox{d}}}}/{V}_{{{\mbox{d0}}}} \sim {(m/{m}_{0})}^{0.23}$, but continue to autorotate up to *m*/*m*_0_ ≈ 1.5. Whereas, the underweighted *A. negundo* is least sensitive to changes in mass, $V_{{{\mbox{d}}}}/{V}_{d0} \sim {(m/{m}_{0})}^{0.06}$, with autorotation for 0.5 ≲ *m*/*m*_0_ ≲ 2.25. The best-fit scaling for the four augmented species is $V_{{{\mbox{d}}}}/{V}_{d0} \sim {(m/{m}_{0})}^{0.12}$, *R*^2^ = 0.80. As a comparison, we also plot $V_{{{\mbox{d}}}}/{V}_{d0} \sim {(m/{m}_{0})}^{0.5}$ with a dashed line from the prediction of Eqs. (1) and (2) for a constant area and note that the flight performance in response to changes in mass of these specimens is considerably different from what is predicted. Overall, samara descent velocity is remarkably robust to mass addition and subtraction. It is clear that the starting point of the individual species is important such that the underweighted samara tested here (*A. negundo*) can withstand the most relative mass addition, whereas the overweighted samara (*A. saccharum*) performs slightly better when mass is reduced. Further, the velocity change is remarkably only ± 15% of the average velocity for mass changes up to ± 70%, revealing their ability to withstand major mass changes and still descend as intended. Mass subtraction, however, does not usher the descent speed retarding benefits predicted by the force balance of Eq. (2) (i.e. velocity reduction values are above ${(m/{m}_{0})}^{0.5}$). Though Varshney et al.^7^ do not report their test species, their results are comparable to our largest seeds (Fig. 8a), with a mass reduction at the nutlet producing *V*_d_/*V*_0_ = 0.75 for *m*/*m*_0_ = 0.471.

While mass alteration does little to influence samara descent velocity, the response in angular velocity is considerably more pronounced. Our *Acer* samaras maintain a relatively stable descent velocity by rotating more rapidly, generating more lift, in response to mass addition. We plot *ω*/*ω*_0_ vs *m*/*m*_0_ in Fig. 8b, where fitting all trials yeilds $\omega /{\omega }_{0} \sim m/{m}_{0}^{0.70}$, *R*^2^ = 0.77. Weighting the smallest samara, *A. buergerianum*, produces the fastest rotation, *ω*/*ω*_0_ ≈ 2 for *m*/*m*_0_ ≈ 1.5. A greater increase in *ω* than *V*_d_ produces lower angles of attack *ϕ* for heavier *Acer* samaras (Fig. S6).

More rapidly rotating samaras descend with a flatter cone angle shown in Fig. 8c such that $\theta /{\theta }_{0} \sim {(m/{m}_{0})}^{-0.48}$, *R*^2^ = 0.71. We posit that the reduction in coning angle for added nutlet mass is the key to keeping *V*_*d*_/*V*_d0_ lower than what would otherwise be expected from Eq. (2). To check this, we plot $\rho {V}_{{{{{{{{\rm{d}}}}}}}}}^{2}A\cos \theta$ vs *mg* in Fig. 8d, where black symbols represent unaltered specimens from each species. Color-coded dashed lines represent the linear correlation of Eq. (2) using individual species data plotted in Fig. 5a, and their length represents the bounds of the original data. The reduction in coning angle enables samaras to outperform naturally heavier individuals within their own species even with an increase in *V*_d_. The overall fit to all our altered trials (solid line) is nearly identical to the data in Fig. 5a (dashed line). Deviations from the samara solid fit line are expected for overweighted and underweighted species (similar to Fig. 5a). Deviations within species (colored dashed lines) are also expected since we altered the nutlet weight. Our results demonstrate that samaras are wonderfully adapted to carry altered masses up to 70% and even to 120% mass increase for underweighted species. Whether this is a genetically beneficial or necessary trait is worthy of future research.

#### Samaras autorotate with severely damaged wings

**Fig. 9:**
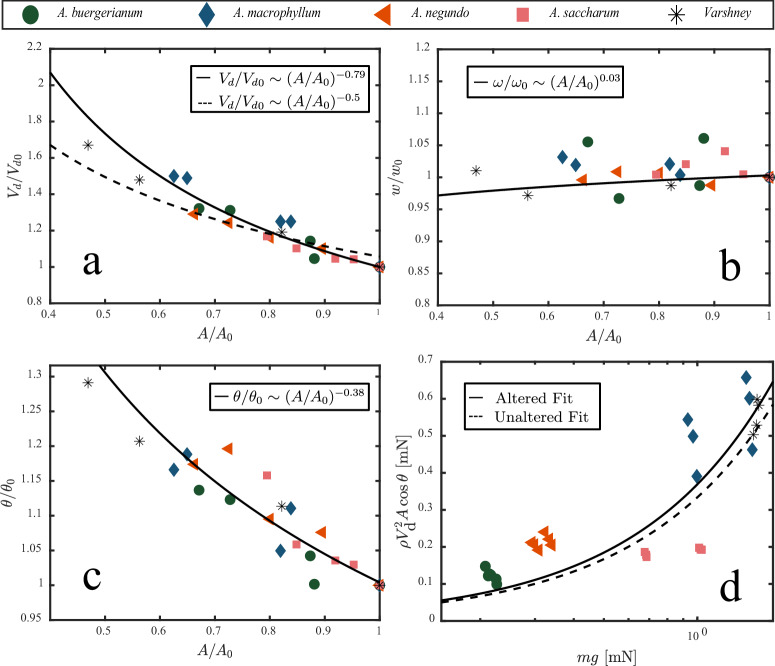
Samaras are robust to wing area ablation. **a** Area ratio *A*/*A*_0_ vs velocity ratio *V*/*V*_0_, **b** area ratio *A*/*A*_0_ vs rotational velocity ratio *ω*/*ω*_0_, **c** area ratio *A*/*A*_0_ vs angular ratio *θ*/*θ*_0_, and **d** weight *mg* to drag.

The long development time of samaras, ~4 months^28^, makes samaras susceptible to herbivores that can damage their wings. Other events such as hail strikes might also induce damage. As with alterations to mass, we can further test Eq. (2) by perturbing samara wing area by ablation, similar to that done by Varshney et al.^7^. In so doing, we probe the robustness of samaras to damage to their thin, brittle wings. The addition of wing area is intractable, but the reduction in area of the wing at a location that is least aerodynamically sensitive, the trailing edge, is straightforward (Methods, Fig. S1). Ablation is done by removing strips of the wing from the trailing edge at ~25%, 50%, and 75% of the chord and done until the samara can no longer stably autorotate in our wind tunnel near *V*_d_/*V*_d0_ ≈ 1.3 − 1.5, at a reduced area *A*/*A*_0_ ≈ 0.6 − 0.8. Such removal of the thin wing has little effect on mass, *m*/*m*_0_ ≥ 0.96. We plot *V*_d_/*V*_d0_ to *A*/*A*_0_ in Fig. 9a. Descent velocity ${V}_{{{{{{{{\rm{d}}}}}}}}}/{V}_{{{{{{{{\rm{d0}}}}}}}}} \sim {(A/{A}_{0})}^{-0.79}$, *R*^2^ = 0.91 is more sensitive to changes in area than predicted by Eq. (2) with all other variables held constant, ${V}_{{{{{{{{\rm{d}}}}}}}}}/{V}_{{{{{{{{\rm{d0}}}}}}}}} \sim {(A/{A}_{0})}^{-0.5}$. Angular velocity is nearly unchanged by reduction in area, $\omega /{\omega }_{0} \sim {(A/{A}_{0})}^{0.03}$, even close to failure, as shown in Fig. 9b. Coning angle *θ* increases with a reduction in area such that $\theta /{\theta }_{0} \sim {(A/{A}_{0})}^{-0.38}$, *R*^2^ = 0.87, with no notable standouts, as shown in Fig. 9c. A hovering samara with an ablated wing is shown in Movie S3.

We plot drag force to weight for reduced wings in Fig. 9d. Across all species, the linear fit to altered area samaras (solid line) and unaltered data in Fig. 5a are similar. We thus conclude that samaras are robust to wing damage and area reduction on their trailing edge. Since we inflict damage on the trailing edges only, our results suggest that our modifications do little to affect the compact leading edge vortex critical for lift generation^17^. Removing wing area from both the leading and trailing edges has been shown to dramatically increase descent velocity as shown by Varshney et al.^7^ and matches the trends in our data shown in Fig. 9.

#### Altered samaras obey wing loading predictions

**Fig. 10:**
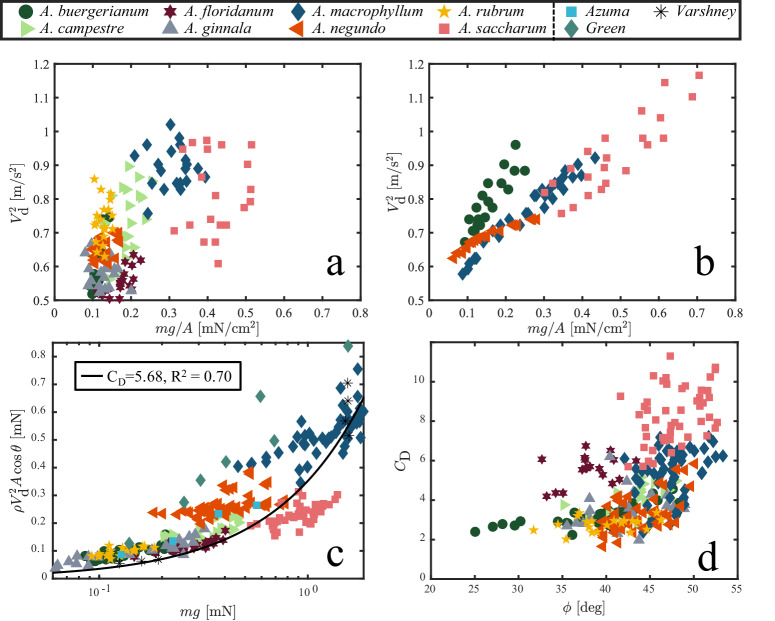
Descent relations for unaltered and altered specimens, and data from literature. Relation between the square of decent velocity and wing loading on (**a**) unaltered (from Fig. 3a) (**b**) altered samaras. Alterations include mass addition and subtraction. **c** Drag coefficient *C*_D_ versus samara weight *mg* for all altered and unaltered specimens in this study. **d** Drag force versus *mg* for all samaras in this study and select data from literature^7,11,15^.

We discuss in “Aerodynamic drag unifies samara species” that a simple drag model, given by Eq. (2), is superior to Eq. (1) for describing the descent characteristics across samara species when numerous, unaltered maple samaras are used. The data presented in Fig. 3a, is presented again in Fig. 10a with adjusted axes. We observe a weak correlation between ${V}_{{{{{{{{\rm{d}}}}}}}}}^{2}$ and *mg*/*A* for unaltered specimens. However, specimens in which mass is added and subtracted well obey ${V}_{{{{{{{{\rm{d}}}}}}}}}^{2} \sim mg/A$, as shown in Fig. 10b. The comparison of panels (a) and (b) reinforces that maple samaras are robust to changes in mass, but more strikingly, that the performance of an individual specimen is inherent to the individual and more complex than a bare *mg*/*A* value can predict. We posit this is due to the small variations between specimens. Despite how well changes to descent velocity by mass alteration are predicted by wing loading, we observe that drag force and weight are more highly correlated. We plot all our experimental drag force values, altered and unaltered, alongside values from literature^7,11,15^ in Fig. 10c. Now, our drag model in Eq. (2) achieves an *R*^2^ = 0.70, with an average *C*_D_ = 5.68. The remarkable similarity in the slopes in Fig. 5a and Fig. 10c underscores the robustness of our model. Perhaps the only advantage of Eq. (1) over Eq. (2) is a quick estimation of velocity change without the need to consider the coning angle.

The relation between drag coefficient C_D_ and angle of attack *ϕ* for all our tests is shown in Fig. 10d. The trend between C_D_ and *ϕ* seemingly agrees with that reported by Lentink et al.; *ϕ* changes very little, typically within 10°, with a large range of *C*_D_^17^. While *C*_D_ increases slightly with *mg*, *ϕ* is functionally independent of *mg*, as shown in Fig. S7.

## Conclusions

Our comprehensive study elucidates the remarkable aerodynamic resilience of *Acer* samaras across varying species, demonstrating their robustness to morphological alterations. By challenging previous wing-loading approaches and employing a classical aerodynamic drag model (Eq. (2)), we show that *Acer* samara adaptive capabilities can sustain autorotation and stable descent velocities despite significant changes in mass and wing area. In fact, they can double their weight and suffer only a 15% increase in descent velocity! Further, they can withstand up to a 40% loss of their trailing wing area and still maintain relatively similar rotation and decent velocities. Our findings not only advance understanding of *Acer* samaras dynamics but also contribute valuable insights to the broader implications of morphological diversity and environmental adaptability in nature. Through meticulous experimentation and analysis, this research underscores the intricate balance between form and function in the evolutionary design of seed dispersal mechanisms.

## Methods

### Mass and area measurements

Samaras from *A. buergerianum* Miq., *A. rubrum* L., *A. ginnala* Maxim., *A. floridanum* Chapm., *A. saccharum* Marhs., *A. campestre* L., *A. negundo* L., and *A. macrophyllum* Pursh. are sourced from online platforms and collected from local trees in Knoxville, TN. The seeds are collected after dispersing naturally. We assessed specimens for damage to exclude visibly damaged seeds from the study. A total of 20 undamaged seeds per species are selected for measurement and subsequent experimentation. An average sample from each species is shown in Fig. 1.

Mass is measured with a Sartorius Secura 225D–1S analytical balance with 0.0001-g resolution. The mass of the samara is measured just before flight trials. Samaras undergoing mass addition have their nutlets dunked into liquefied wax. Mass subtraction is done by clipping small portions from the nutlet. Seeds undergoing reduced area have the trailing edge of the wing excised while maintaining the original span.

### Dynamic measurements and filming

A schematic of our experimental flight setup is shown in Fig. 2c. Two wind tunnels were constructed specifically for studying samara flight, with dimensions of 15 × 15 cm and 20 × 20 cm. The construction of two tunnels is essential to provide sufficient room for the larger seeds to fly without encountering the boundaries of the smaller tunnel. The larger wind tunnel accommodated *Acer Macrophyllum* and *Acer Negundo* specimens, in addition to accommodating samaras with added mass and reduced area. A 20.3-cm inline duct fan pushes air into the bottom of the tunnel by way of a flexible duct and through 2.5 cm of aramid honeycomb with a 3.2-mm cell diameter that laminarizes flow^29^. Fan speed is controlled by an ITECH IT-M7721 AC power supply. Wind speed *V*_d_ is measured by a Koselig KI-10001 anemometer with 0.01-m/s precision. A Photron NOVA S6 camera captures samara hovering at 2000 fps.

Samaras are released into wind tunnels by hand. Wind speed is adjusted to facilitate stable hovering such that samaras do not ascend or descend > 1 cm per 7 revolutions. The coning angle is determined by averaging three angle measurements taken during seven complete rotations. Angular velocity is determined by measuring the time taken for the samara seed to complete one rotation, and another, after seven subsequent rotations.

### Statistics and reproducibility

We employ the MATLAB box chart tool to visualize the experimental data distributions, including the minimum, maximum, average, and first and third quartiles. These values were presented in the colored boxes of Fig. 4 and Fig. 6. All wind tunnel trials are comprised of three replicates. All reported averages in tables and plots derived from wind tunnel experiments have standard deviations smaller than the marker size.

### Reporting summary

Further information on research design is available in the Nature Portfolio Reporting Summary linked to this article.

### Supplementary information

Supplementary Information

Description of Additional Supplementary Files

Movie S1

Movie S2

Movie S3

Reporting Summary

## Data Availability

All tabulated data used to make figures are available in perpetuity at https://osf.io/3pzu8/. Raw videos will be made available upon request.
