# Supplementary material for: Maple samara flight is robust to morphological perturbation and united by a classic drag model
Source: Commun Biol. 2024 Mar 1;7:248. doi: 10.1038/s42003-024-05913-3 (PMC10907639; doi:10.1038/s42003-024-05913-3)
Supplement: Supplementary file 1 — Supplementary Information [file 42003_2024_5913_MOESM1_ESM.pdf]

Online Supplement for *Maple samara flight is robust to morphological perturbation and united by a classic drag model*

1 Supplementary movie captions

Movie S1: *A. negundo* samara hovering unaltered at 0.79 m/s (left) and with 19 mg of mass addition at 0.82 m/s (right). The samara on the right has $m/m_0 = 1.34$ and $V/V_0 = 1.04$. Slowed 67×.

Movie S2: *A. macrophyllum* samara hovering unaltered at 0.92 m/s (left) and with 34 mg of mass reduction at 0.86 m/s (right). The samara on the right has $m/m_0 = 0.65$ and $V/V_0 = 0.93$. Slowed 67×.

Movie S3: *A. buergerianum* samara hovering unaltered at 0.77 m/s (left) and with 92 mm^2 of area reduction at 0.88 m/s (right). The samara on the right has $A/A_0 = 0.68$ and $V/V_0 = 1.14$. Slowed 67×.

2 Supplementary figures

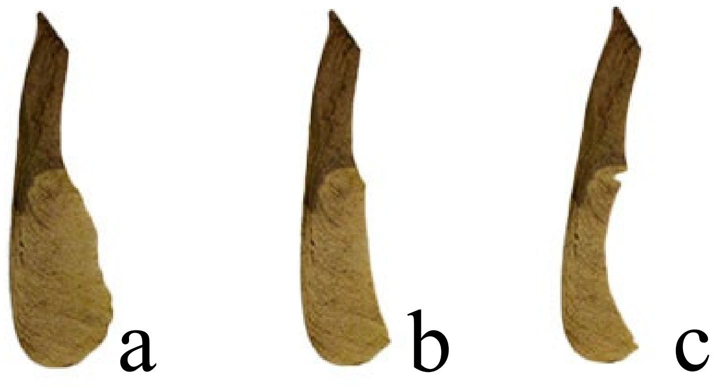

Figure S1: A series of area reductions from an *A. Negundo* samara. (a) unaltered, (b) cut once, and (c) cut again.

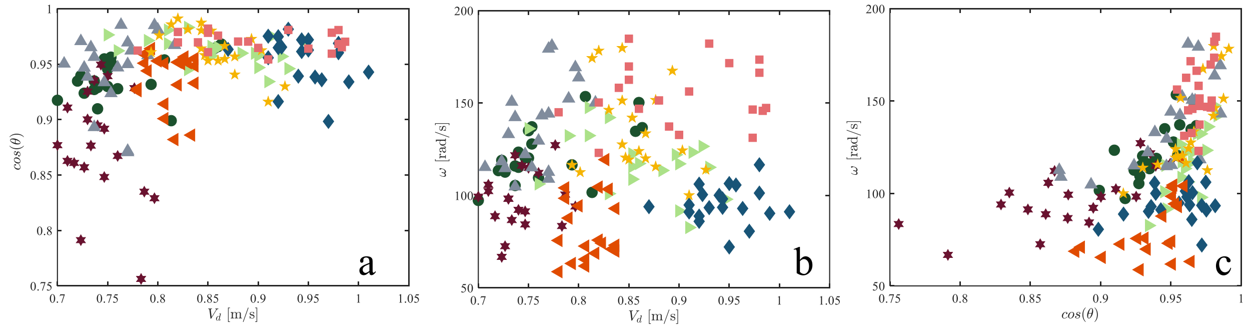

Figure S2: Relationships between dynamic variables (a) V_d vs $cos(\theta)$, (b) V_d vs ω (c) $cos(\theta)$ vs ω

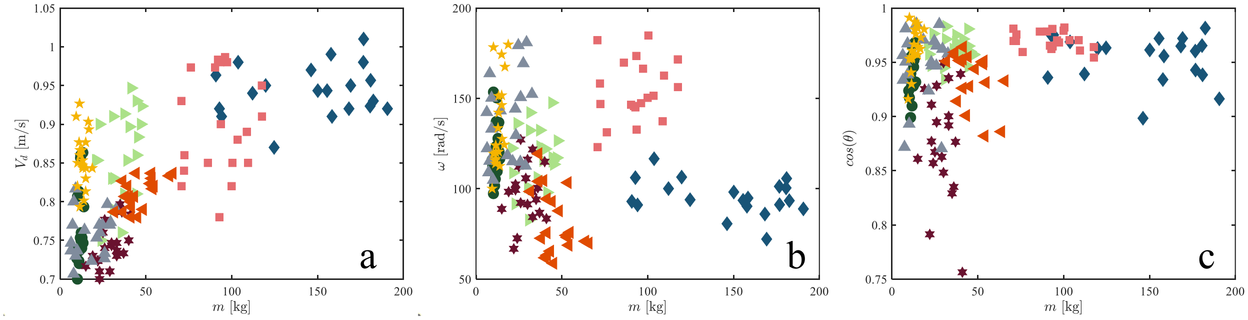

Figure S3: Relationships between mass and dynamic variables (a) m vs V_d, (b) m vs ω (c) m vs $cos(\theta)$

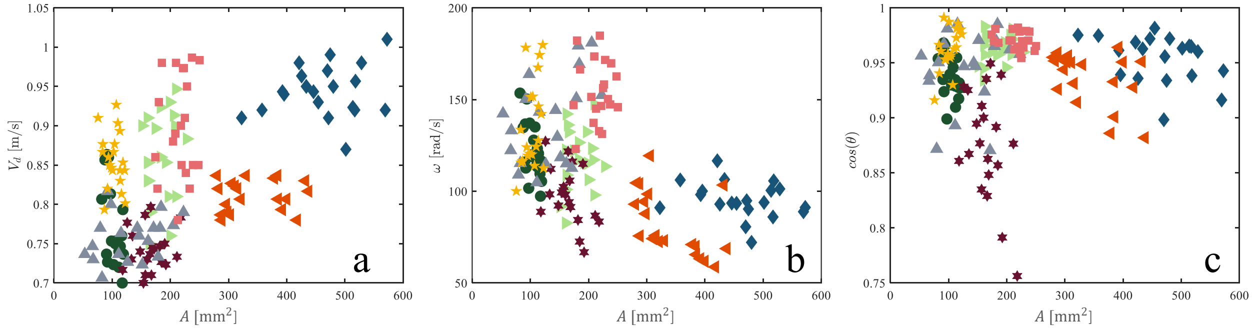

Figure S4: Relationships between area and dynamic variables (a) A vs V_d, (b) A vs ω (c) A vs $cos(\theta)$

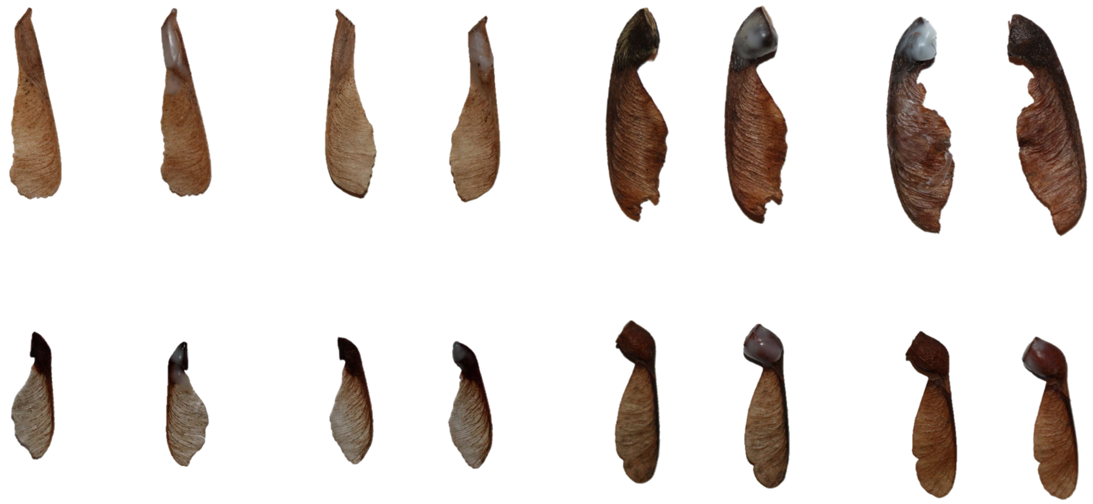

Species	*Percent Change*		
A. negundo	+ 1.3	+1.4	+1.9
A. ginnala	+3.5	+3.6	+3.2
A. saccharum	+3.1	+3.2	+3.6
A. macrophyllum	+4.8	+3.5	+2.7

Figure S5: Increased mass on samara seeds. The images showcase the seeds before and after the mass augmentation process. The table presents the corresponding percentage changes in area for each seed.

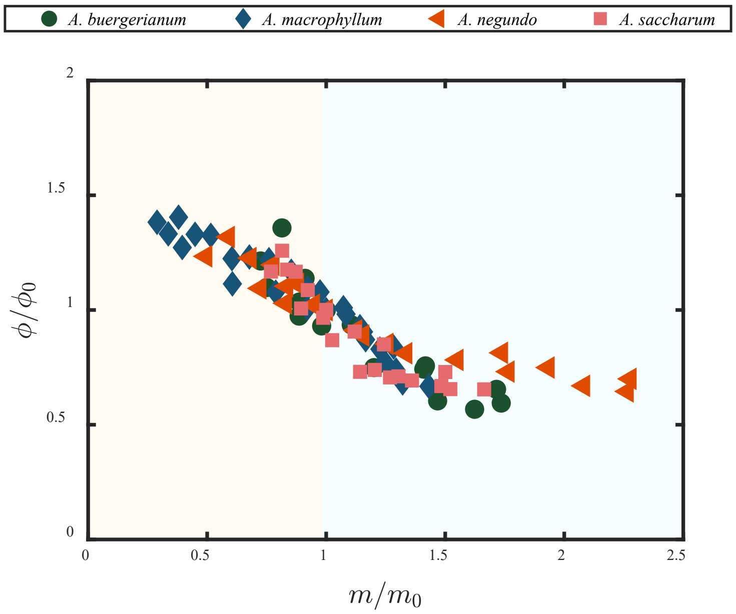

Figure S6: Reduced angle of attack ϕ/ϕ_0 vs reduced mass m/m_0

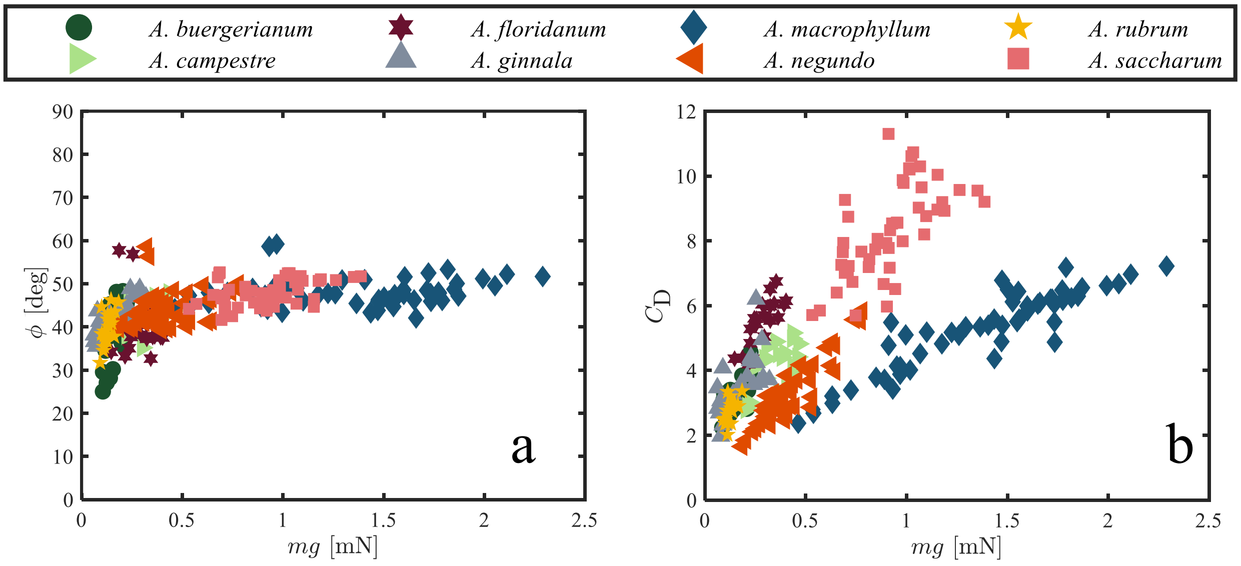

Figure S7: Relationship between weight mg and angle of attack ϕ **(a)** Relationship between weight mg and C_D
