# Supplementary material for: Maple samara flight is robust to morphological perturbation and united by a classic drag model
Source: Commun Biol. 2024 Mar 1;7:248. doi: 10.1038/s42003-024-05913-3 (PMC10907639; doi:10.1038/s42003-024-05913-3)
Supplement: Supplementary file 2 — Description of Additional Supplementary Files [file 42003_2024_5913_MOESM2_ESM.pdf]

Description of Additional Supplementary Files

File name: Movie S1
Description: A. negundo samara hovering unaltered at 0.79 m/s (left) and with 19 mg of mass addition at 0.82 m/s (right). The samara on the right has m/m0 = 1.34 and V /V0 = 1.04. Slowed 67×.

File name: Movie S2
Description: A. macrophyllum samara hovering unaltered at 0.92 m/s (left) and with 34 mg of mass reduction at 0.86 m/s (right). The samara on the right has m/m0 = 0.65 and V /V0 = 0.93. Slowed 67×.

File name: Movie S3
Description: A. buergerianum samara hovering unaltered at 0.77 m/s (left) and with 92 mm2 of area reduction at 0.88 m/s (right). The samara on the right has A/A0 = 0.68 and V /V0 = 1.14. Slowed 67×.
